# Dissecting domains necessary for activation and repression of splicing by muscleblind-like protein 1

**DOI:** 10.1186/1471-2199-14-29

**Published:** 2013-12-27

**Authors:** Christopher Edge, Clare Gooding, Christopher WJ Smith

**Affiliations:** 1 Department of Biochemistry, University of Cambridge, Tennis Court Road, Cambridge CB2 1QW, UK

## Abstract

**Background:**

Alternative splicing contributes to the diversity of the proteome, and provides the cell with an important additional layer of regulation of gene expression. Among the many RNA binding proteins that regulate alternative splicing pathways are the Muscleblind-like (MBNL) proteins. MBNL proteins bind YGCY motifs in RNA via four CCCH zinc fingers arranged in two tandem arrays, and play a crucial role in the transition from embryonic to adult muscle splicing patterns, deregulation of which leads to Myotonic Dystrophy. Like many other RNA binding proteins, MBNL proteins can act as both activators or repressors of different splicing events.

**Results:**

We used targeted point mutations to interfere with the RNA binding of MBNL1 zinc fingers individually and in combination. The effects of the mutations were tested in assays for splicing repression and activation, including overexpression, complementation of siRNA-mediated knockdown, and artificial tethering using MS2 coat protein. Mutations were tested in the context of both full length MBNL1 as well as a series of truncation mutants. Individual mutations within full length MBNL1 had little effect, but mutations in ZF1 and 2 combined were more detrimental than those in ZF 3 and 4, upon splicing activation, repression and RNA binding. Activation and repression both required linker sequences between ZF2 and 3, but activation was more sensitive to loss of linker sequences.

**Conclusions:**

Our results highlight the importance of RNA binding by MBNL ZF domains 1 and 2 for splicing regulatory activity, even when the protein is artificially recruited to its regulatory location on target RNAs. However, RNA binding is not sufficient for activity; additional regions between ZF 2 and 3 are also essential. Activation and repression show differential sensitivity to truncation of this linker region, suggesting interactions with different sets of cofactors for the two types of activity.

## Background

Pre-mRNA splicing is a critical part of mRNA maturation, and alternative splicing is a well established method of generating diversity and exerting control over the proteome. It is now recognised that the vast majority of transcripts are alternatively spliced, allowing production of many protein isoforms from a single gene (for review see [1]). The process is controlled so that certain isoforms are restricted to specific cell types, developmental stages, or conditions [2,3]. Alternative splicing is controlled in large part by a variety of a protein factors which can positively or negatively influence splicing at adjacent splice sites. Early investigations suggested that proteins of the SR family generally act as splicing activators, while proteins of the hnRNP family typically act as repressors. More recent global analyses of the activities of RNA binding proteins has indicated that many of them show both activator or repressor activity, depending on the site at which they bind to the target pre-mRNA [4].

Loss of regulation of alternative splicing can lead to a variety of diseases, including Myotonic Dystrophy (DM1), which is caused by expansions of CUG repeats, which bind and sequester muscleblind like (MBNL) proteins [5]. MBNL proteins normally control the transition from embryonic to adult isoforms of a sub-set of muscle-specific proteins in heart and skeletal muscle cells [6-8]. In DM1, embryonic isoforms of important muscle proteins are expressed, which causes the various clinical symptoms [9,10]. For example, myotonia is casued by deregulation of a MBNL-controlled splicing event in the skeletal muscle chloride channel (CLCN1) [11].

**Figure 1:**
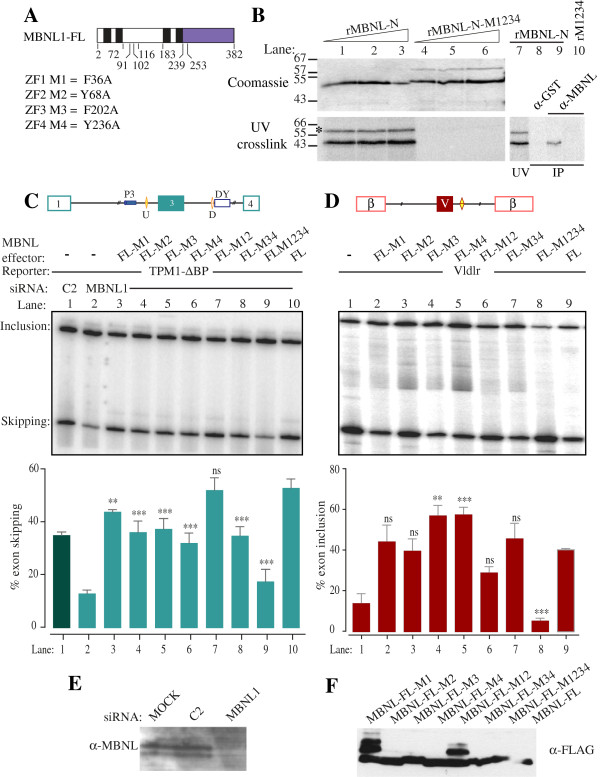
**Effects of RNA binding mutations on MBNL1 splicing activity. A**. Schematic representation of MBNL1. Zinc fingers are shown in black, the C-terminus in purple. Amino acid positions of deletion boundaries mutants and ZF domain inactivating mutations are indicated. The 382 aa MBNL1 isoform lacks sequences corresponding to exons 7 and 9. **B**. Comparison of wild type MBNL-N and MBNL-N-M1234 UV crosslinking to RNA. Upper panel, Coomassie blue stained gel; lower panel UV crosslinking. RNA used for crosslinking encompassed Tpm1 exon 3 and both upstream and downstream MBNL elements. The identity of crosslinked MBNL (lanes 1-3) was established by immunoprecipitation with anti-MBNL1 (lane 9) but not anti-GST antibodies (lane 8). The asterisked band is a contaminant that was not immunoprecipitated by anti-MBNL1; it does not correspond to the higher molecular weight contaminant in the Coomassie stained samples of mutant protein (lanes 4–6). **C**. Effects of MBNL1 knockdown and overexpression upon Tpm1 splicing in HeLa cells. The cartoon depicts the ΔBP minigene; the U and D MBNL binding elements and P3 and DY pyrimidine tracts are indicated. The minigene was co-transfected with control (C2, lane 1) or MBNL1 siRNAs (lanes 2–10), and with wild-type MBNL1 (lane 10) or MBNL1 mutants in the indicated ZF domains (lanes 3–9). Values significantly different from FL wild type MBNL1: **, P < 0.01; *** P < 0.001; ns, not significant. Values for the M1234 mutant are statistically significant, but lack of protein expression (panel F) prevents meaningful conclusions from being drawn. **D**. Effects of MBNL1 overexpression upon Vldlr splicing in HeLa cells. The cartoon depicts the Vldlr minigene; the MBNL1 binding site is indicated by the yellow diamond. The minigene was transfected alone (lane 1), with wild-type MBNL1 (lane 9) or mutants in ZF domains (lanes 2–8). **E**. Western blot of MBNL1 in mock transfected, control(C2) or MBNL1 siRNA treated cells. **F**. Anti-FLAG western blot showing expression of MBNL1 constructs from panels **C** and **D**.

MBNL is a four zinc-finger (ZF) containing protein (of the type CX_7_CX_4-6_CX_3_H). The ZF domains are arranged in two tandem arrays in the N-terminal part of the protein (Figure 1A). The RNA binding faces in each didomain are arranged back-to-back, creating a predicted anti-parallel alignment of RNA binding to adjacent ZFs [12,13]. SELEX experiments have determined the optimal MBNL binding sequence to consist of multiple YGCY motifs [14], explaining the binding to CUG expansions. By using U-tracts with two GC steps and manipulating the spacing between them, it has been shown that MBNL can bind the two sites with as little as a 1 nt spacer separating them, or in a second binding conformation with a spacer of around 17 nt [15], suggesting multiple modes of RNA-protein interaction. The published crystal structures of MBNL1 ZF domains [13] shows how the two domains in the ZF34 tandem array interact with the RNA. Key aromatic residues in ZF3 and 4 (F202 and Y236) intercalate between the bases of the GC step, while specific hydrogen bonds from the GC bases to side chains in the protein partly explain the binding specificity of MBNL-1.

The MBNL1 gene is comprised of 12 exons, 10 of them protein coding, with the ZFs encoded by exons 2–6. Extensive alternative splicing of exons encoding the linker between ZFs 2 and 3, and the C-terminal end of the protein leads to multiple functionally distinct protein isoforms [16]. Structure-function analyses of MBNL1 and 3 have been performed by generating N- and C– terminal truncations and analysing the effect on splicing regulation. In this analysis, the regions of MBNL required for splicing repression and activation differed. Activation required the entire linker sequence between ZF 2 and 3, while repression required only a small N-terminal portion of the linker [17]. A second structure-function analysis involved targeted mutations to impair RNA binding by the different ZF domains, and analysis of the consequences upon MBNL-repressed and MBNL-activated events [18]. Although activity is usually linked to RNA binding, there is a subset of events where the affinity of MBNL for the RNA is not correlated with activity.

Artificial recruitment systems have been used to great effect to analyze the function of splicing factors and other RNA binding proteins. This method involves expressing the protein of interest as a fusion with a heterologous RNA binding protein, such as MS2 coat-protein, and replacing the normal binding site on the target RNA with an MS2 binding site. This circumvents the normal mode of RNA binding and allows the dissection of splicing activator or repressor domains. This approach has been used to investigate SR proteins [19] hnRNP and other RNA binding proteins including hnRNP A1 [20], PTB [21], MBNL1 [22], RbFOX [23] and hnRNPL [24].

Here we use targeted mutations to disrupt RNA binding by individual ZF domains of MBNL1 combined with larger deletions to analyse the splicing activation and repression function of MBNL1 in both MS2-tethered and non-tethered splicing assays. We find that full length MBNL1 is remarkably tolerant of mutation to individual ZFs or pairs of ZFs in a simple cotransfection assay. However, in MS2 tethering assays of the N-terminal part of MBNL1 containing the four ZF domains, mutation of ZF3 and 4 has no effect on splicing repression, but mutation of ZF1 and 2 is highly deleterious. In contrast, for activation no mutations or pairs of mutations drastically reduce activity. When artificially recruited, only the first two zinc fingers plus a small N-terminal portion of the linker sequence between ZF 2 and 3 is required for repression, whereas for activation the whole linker sequence is needed even though this region plays no part in RNA binding. For both activation or repression, disruption of RNA binding by ZFs 1 and 2 is highly deleterious for activity. Our results further highlight the distinct requirements of different regions of MBNL1 for splicing repression and activation.

## Results

### Effect of MBNL RNA binding mutations on MBNL-regulated splicing events

Based on high resolution structures of the TIS11d [25] and MBNL proteins [12,13] we designed point mutations in each MBNL zinc finger that would disrupt RNA binding, without severely altering the overall fold and structure of the domain. We targeted conserved aromatic residues F36, Y68, F202 and Y236 in ZF 1–4 respectively, and mutated them to alanine (Figure 1A). The mutations were introduced individually, in combinations in the two di-domains (MBNL-FL-M12 and -M34) and into all four ZF domains simultaneously. Similar mutations have since been reported by others [18,26]. In order to confirm that the mutations disrupt RNA binding, recombinant MBNL1 aa 2–253 was produced with all four zinc fingers mutated and compared to wildtype protein in UV crosslinking assays. While the wild-type crosslinked to the RNA the mutant did not (Figure 1B, lower panel).

We next tested the effects of the MBNL ZF mutants in assays for splicing repression and splicing activation by MBNL1 in HeLa cells. To test splicing repressor activity we used a *Tpm1* minigene with a point mutation of the branch point of exon 3, which increases exon 3 skipping in HeLa cells [22,27]. This minigene responds modestly to simple overexpression of MBNL1. However, upon knockdown of MBNL1 (Figure 1E) exon skipping is reduced substantially (from 35 to 13%, Figure 1C, lanes 1, 2); complementation with overexpressed MBNL1 restores exon skipping to 53% (Figure 1C, lane 10). As a model MBNL-activated exon we used a minigene construct containing a Vldlr exon flanked by globin exons [10], which responds to MBNL1 overexpression by increasing exon inclusion from 14 to 39% (Figure 1D, lanes 1, 9). Note that in order to facilitate comparison of the repressor and activator activities of MBNL1 mutants, we refer throughout to percentage exon skipping of the repressed *Tpm1* exon but percentage exon inclusion of the activated *Vldlr* exon.

Compared to wild type MBNL1, all of the ZF domain single point-mutants had moderately reduced repressor activity, producing exon skipping levels of 32-44% (Figure 1C, lanes 3–6), as did the combined ZNF 3 and 4 mutant (lane 8). Surprisingly, the mutant with combined mutations in ZF 1 and 2 was as active as wild type MBNL1 (lane 7), despite being expressed at similar levels to the other constructs (Figure 1F). The mutant with all four ZF domains impaired showed no activity (Figure 1C, lanes 2 and 9). However, this mutant was consistently expressed at lower levels than the other constructs (Figure 1F), preventing strong conclusions about its activity. We noted that the MBNL proteins with mutations in ZF1 (MBNL-FL-M1 and MBNL-FL-M12) consistently showed the presence of additional slower migrating bands that were detected with FLAG antibodies (Figure 1F). We do not know the explanation for these additional bands, or whether they represent an active fraction of protein. It is therefore possible that higher total levels of active proteins with the M1 mutation might partially mask loss of activity induced by the mutation.

Mutations in ZF1 or 2 had no significant effect upon the ability of MBNL1 to activate Vldlr splicing (Figure 1D, lanes 2,3,9), while mutations in ZF 3 or 4 individually caused a small but significant increase in activity (lanes 4,5). Double mutations of ZF 3 and 4 or 1 and 2 combined were also without significant effect (lanes 6,7), although the 12 mutant was significantly less active than 34 (P < 0.05). Only the quadruple ZF1-4 mutant showed significantly lower activity than WT MBNL1 (lane 8), but again no firm conclusions could be drawn due to the much lower expression levels of this mutant (Figure 1F).

Taken together, the preceding data indicated that both repressor and activator activities of MBNL1 are remarkably tolerant of mutations that impair RNA binding of individual ZF domains, and even mutations of both ZFs within a didomain have limited effects.

### MS2 tethering of MBNL1 activation and repression domains

**Figure 2:**
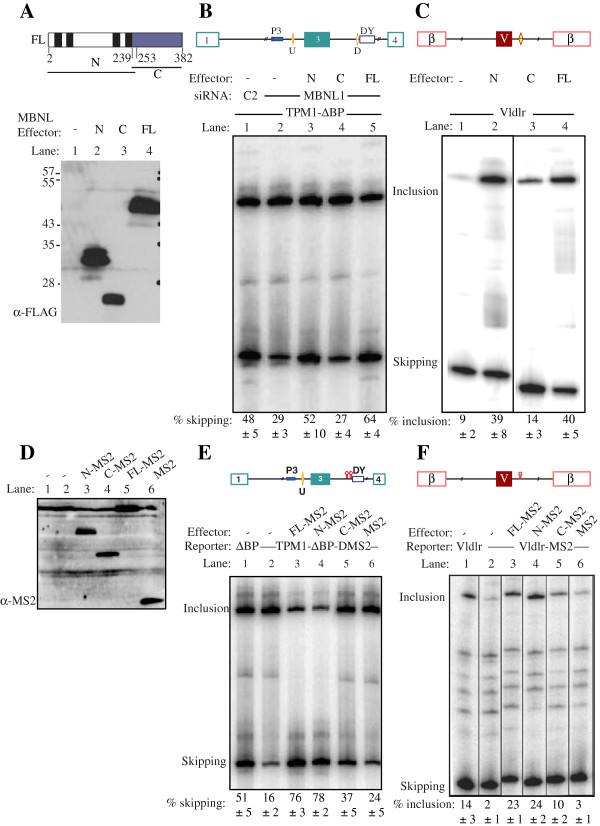
**Activity of MBNL1 N and C-terminal domains in splicing repression and activation. A**. Schematic of the regions of MBNL1 used in the experiments in this figure (top). Lower panel, anti-FLAG western blot showing expression of MBNL proteins. **B**. The Tpm1 ΔBP minigene was co-transfected with control (C2, lane 1) or MBNL1 targeting siRNAs (lanes 2–5). In addition, full-length MBNL1 (lane 5) or the MBNL1 N- or C-terminal domains (lanes 3–4) were cotransfected along with the MBNL1 siRNA. **C**. The Vldlr minigene was transfected alone (lane 1) or with full-length MBNL1 (lane 4) or the MBNL1 N- or C-terminal domains (lanes 2, 3). **D**. Anti-MS2 western blot of HeLa cells transfected with MS2 alone, lane 6, or MBNL1-MS2 fusion proteins: full length, lane 5; MBNL1 C-terminus, lane 4, MBNL1 N-terminus, lane 3; mock transfections lanes 1, 2. **E**. Schematic of the Tpm1 ΔBP DMS2 minigene in which the downstream MBNL site is replaced by a pair of MS2 hairpins (upper panel). Lower panel, RT-PCR of HeLa cells transfected with Tpm1 ΔBP (lane 1) or Tpm1 ΔBP DMS2 (lanes 2–6). Cotransfections with MS2 alone (lane 6) or the indicated MBNL1-MS2 fusion proteins (lanes 3–5). **F**. Schematic of the Vldlr-MS2 minigene in which the downstream MBNL site is replaced by an MS2 hairpin (upper panel). Lower panel, RT-PCR of HeLa cells transfected with Vldlr (lane 1) or Vldlr-MS2 (lanes 2–6). Cotransfections with MS2 alone (lane 6) or the indicated MBNL1-MS2 fusion proteins (lanes 3–5).

We next compared the activities of deletion mutants of MBNL1 in simple cotransfection and tethered function assays (Figure 2). Consistent with previous data [22] in the knockdown/complementation assay with the *Tpm1* reporter, the N-terminal region of MBNL1 (aa 2–253) had similar repressor activity to the full length protein (Figure 2B, lanes 3,5). In contrast, a C-terminal fragment of MBNL1 (aa 239–382) had no activity (Figure 2B, lane 4). Similar effects were seen with the Vldlr reporter; the N-terminal fragment had indistinguishable activity to full length MBNL1 (Figure 2C, lanes 2,4), while the C-terminal fragment was devoid of activator activity (lane 3), despite being expressed to similar levels (Figure 2A).

As reported previously [22] replacement of the downstream MBNL1 binding element in *Tpm1* with a binding site for MS2 coat protein led to a ~3-fold decrease in exon skipping (Figure 2E, lanes 1,2). Addition of MS2 coat protein had little effect (lane 6), while fusion proteins of MS2 with full length MBNL1 or just the N-terminal region led to high levels of exon skipping (lanes 3,4). In contrast, the C-terminal region of MBNL1 fused to MS2 had a significant, but much more modest effect than full length MBNL1-MS2 (lane 5). Replacement of the reported MBNL1 binding site containing two GC motifs downstream of the Vldlr exon with a single MS2 site reduced exon inclusion from 14% to 2% (Figure 2F, lanes 1,2), consistent with the activity of this element as an MBNL-dependent splicing enhancer in mouse embryonic fibroblasts [10]. Co-transfection with MS2 protein had no effect (lane 6), while full length MBNL1-MS2 restored exon inclusion levels (lane 3). As in the repression assay, the N-terminal of MBNL1-MS2 had full activity, while the C-terminal region had partial activity (lanes 4,5). These data indicate that the N-terminal region of MBNL1 has full activity in simple co-transfection and artificial tethering repression and enhancing assays, while the C-terminal region was inactive in simple cotransfections and had partial activity in tethered assays.

**Figure 3:**
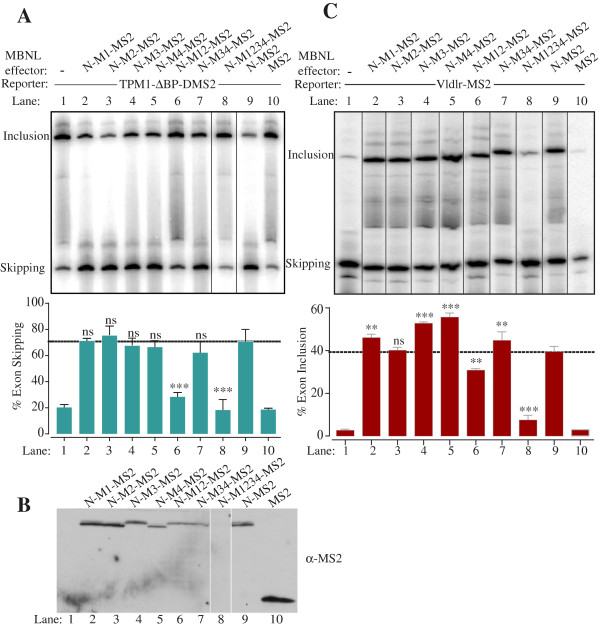
**Effects of RNA binding mutations on tethered MBNL1 repressor and activator function. A**. The Tpm1 ΔBP DMS2 minigene was transfected alone (lane 1), or co-transfected with MS2 (lane 10), or MS2 fused to the N-terminal (aa 2–253) of MBNL1 (lanes 2–9). The MBNL-MS2 fusion proteins were WT (lane 9) or had the indicated ZF mutations (lanes 2–8). The horizontal dashed lines indicate the activity of the wild type N-MS2 (lane 9). Values significantly different from wild type N-MS2 in panels **A** and **C**: **, P < 0.01; *** P < 0.001; ns, not significant. Note that although the values for the M1234 mutant in lane 8 of panels **A** and **C** are statistically significant, the lack of protein expression of the M1234 mutant (panel **B**) means that meaningful conclusions cannot be drawn. **B**. Anti-MS2 western blot of MBNL-MS2 fusion proteins used in panels **A** and **C**. **C**. The Vldlr MS2 minigene was transfected alone (lane 1), or co-transfected with MS2 (lane 10), or MS2 fused to the N-terminal (aa 2–253) of MBNL1 (lanes 2–9). The MBNL-MS2 fusion proteins were WT (lane 9) or had the indicated ZF mutations (lanes 2–8). The horizontal dashed lines indicate the activity of the wild type N-MS2 (lane 9).

In the artificial tethering assay, the MS2 domain serves to recruit the fusion protein to the regulated RNA, presumably bypassing the RNA-binding function of at least some of the ZF domains. To explore this issue we introduced the RNA binding mutations into the ZF domains of the MBNL-N-MS2 construct (Figure 3). Tethering of the WT MBNL-N-MS2 downstream of *Tpm1* exon 3 increased exon skipping from 20% (lanes 1,10) to 71% (lane 9). Individual mutations in ZF1-4 or combined mutations in ZF3 and 4 had no effect on activity (lanes 2–5, 7). However, combined mutations in ZF1 and 2 drastically reduced activity (lane 6), even though the protein was expressed (Figure 3B). Indeed, exon skipping levels in the presence of the ZF12 mutant were not significantly different from MS2 alone or no cotransfection (lane 6, compared to lanes 1 or 10). The quadruple mutant in ZF1-4 was also inactive, but again the protein was expressed at very low levels (lane 8 and Figure 3B). The complete loss of activity upon ZF12 mutation in the tethered repressor assay is in stark contrast to the more modest effects in the simple cotransfection assay (Figures 1C and 3A). In the tethered activation assay the single mutations in ZF1, 3 and 4, and the combined mutation of ZF3 and 4 led to a modest but significant increase in activity while the ZF2 mutation was without effect (Figure 3C, lanes 2–5,7 compared to 9). Only the dual ZF12 mutant showed decreased activity (lane 6) but the effect was modest compared to the loss of repressor activity.

**Figure 4:**
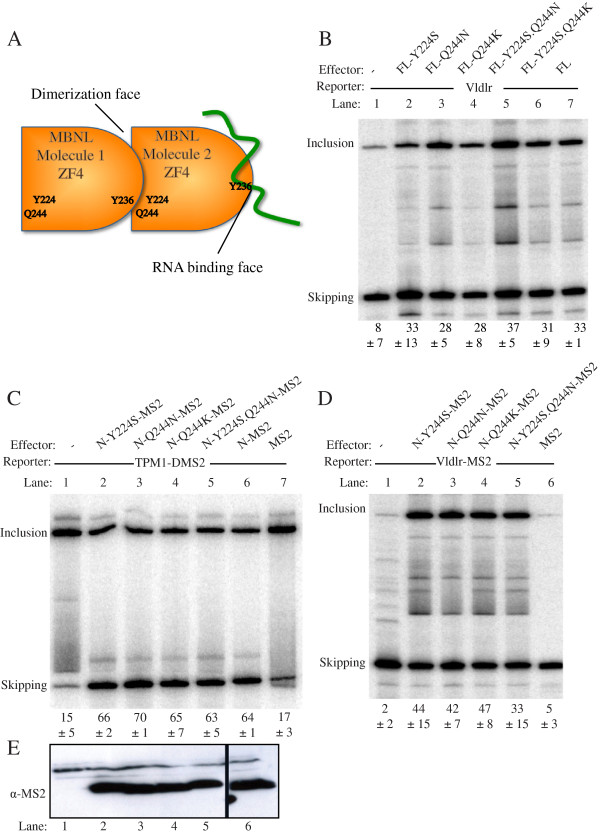
**Mutations in a potential dimerization contact in ZF4 have no effect. A**. Schematic of the potential ZF4-ZF4 dimerization contact identified in MBNL1 crystal structures. The RNA binding face of one ZF4 unit interacts with the opposite face of the second ZF4 unit. Mutation of Y224 and Q244 is predicted to interfere with the potential protein-protein interaction, but not with RNA binding by ZF4. **B**. The Vldlr minigene was transfected alone (lane 1), or with MBNL1 FL (lanes 2–7). The MBNL1 was WT (lane 7) or carried the indicated ZF4 mutations. **C**. The Tpm1 ΔBP DMS2 minigene was transfected alone (lane 1), or co-transfected with MS2 (lane 7), or MS2 fused to the N-terminal (aa 2–253) of MBNL1 (lanes 2–6). The MBNL-MS2 fusion proteins were WT (lane 6) or had the indicated ZF4 mutations (lanes 2–5). **D**. The Vldlr MS2 minigene was transfected alone (lane 1), or co-transfected with MS2 (lane 6), or MS2 fused to the N-terminal (aa 2–253) of MBNL1 (lanes 2–5). The MBNL-MS2 fusion proteins had the indicated ZF mutations (lanes 2–5). **E**. Anti-MS2 western blot of MBNL1-MS2 fusion proteins corresponding to lanes 1–6 of panel **C**.

MBNL1 is thought to dimerize through its C terminus [16,28]. However, the crystal structure of ZF34 revealed a dimerization contact involving the RNA binding face of ZF4 in one subunit, with the reverse face of ZF4 in the other subunit [13]. We tested the effects of individual or combined mutations in Tyrosine 224 (Y224S) and Glutamine 244 (Q244N), which are predicted to impair the potential dimerization contact, but not RNA binding (Figure 4A). These mutations had no effect upon the tethered repressor (4C) or activator (Figure 4D) activities of MBNL-N-MS2, or on the direct activation of Vldlr by full length MBNL1 (Figure 4B). These results suggest that the observed crystal contact between MBNL1 subunits is not important for MBNL1 function.

### MBNL1 binding to RNA species from MBNL-regulated exons

**Figure 5:**
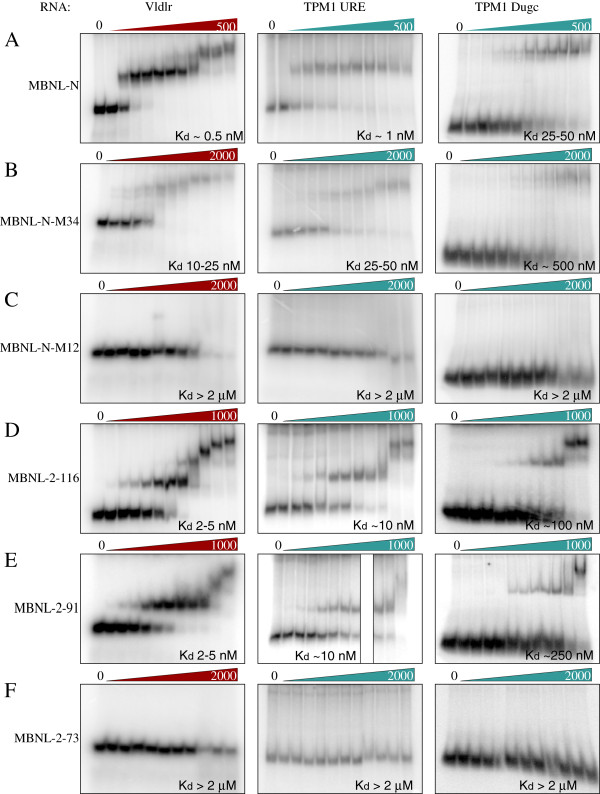
**MBNL binding to Vldlr and Tpm1 RNAs.** RNA binding was assessed by native gel electrophoretic mobility shift assay. RNAs are the MBNL-responsive element from Vldlr (left panels), the upstream MBNL binding site of Tpm1 exon 3 (middle panels), and the downstream MBNL binding site of Tpm1 exon 3 (right panels). Recombinant proteins used were: **A**, MBNL-N, the wild-type MBNL construct comprising amino acids 2–253 and containing all four zinc fingers, **B**, MBNL-N-M34, with ZF34 mutated, **C**, MBNL-N-M12 with ZF12 mutated, **D**, MBNL-2-116 **E**, MBNL-2-91 **F**, MBNL-2-72. Increasing protein concentrations are indicated by the wedges above each panel. Protein concentrations were 0, 0.1, 0.5, 1, 2, 5, 10, 25, 50, 100, 250, 500 nM for panel A, 0, 0.1, 0.5, 1, 2, 5, 10, 25, 50, 100, 250, 1000 nM for panels D and E, and 0, 1, 2, 5, 10, 25, 50, 100, 250, 500, 1000, 2000 nM for panels B, C and F. Estimated K_d_’s are indicated in the lower right corner of each panel.

Having investigated the role of the MBNL1 ZF domains in splicing repression and activation, we next tested the binding of MBNL1-N to RNAs containing the MBNL binding elements of Vldlr and Tpm1 by electrophoretic mobility shift assay (Figure 5). We compared binding of WT MBNL1-N with the mutants in ZF12 (M12) and ZF34 (M34). WT MBNL1 bound to the Vldlr and upstream Tpm1 elements, Tpm1 URE, with Kd in the 0.5 – 1 nM range, while binding to the downstream Tpm1 element Tpm1 Dugc, was approximately 10-fold lower affinity (Figure 5A, K_d_ 25–50 nM). With the Vldlr RNA a second binding event was also observed with a much lower affinity; we observed no additional binding events to either of the Tpm1 elements, even though their length is sufficient to accommodate multiple binding sites [15]. Mutation of ZF34 reduced the affinity of binding to all three RNAs by about ~20-fold (Figure 5B). In contrast, the effects of mutations in ZF12 were far more drastic; no stable complexes were observed on any of the RNAs, even when up to 2 μM MBNL protein was used (Figure 5C). Thus, the N-terminal ZF12 domains are more important for both binding to Tpm1 and Vldlr RNAs, as well as for tethered activity.

### MS2 tethering of MBNL1 truncations

**Figure 6:**
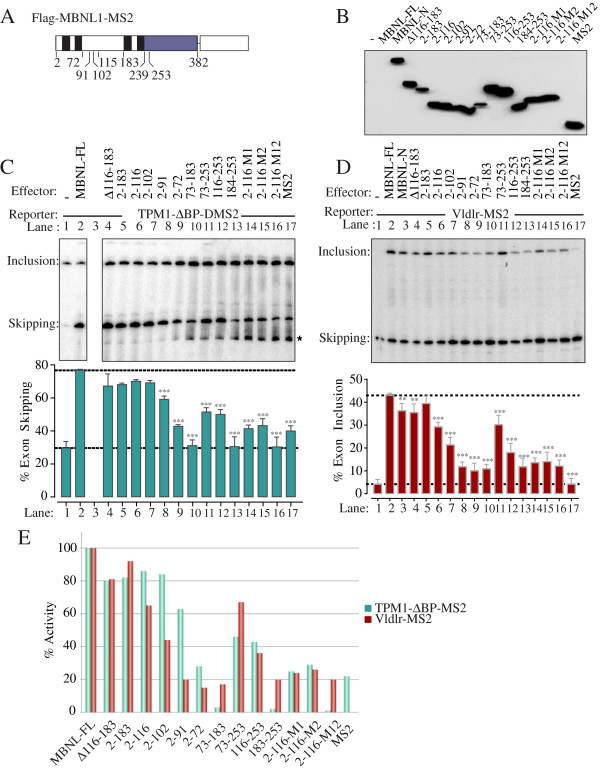
**Comparison of MBNL1 deletions and point mutations upon tethered repression and activation. A**. Schematic of the full-length MBNL1-MS2 construct with boundaries of various deletions indicated. **B**. Anti-MS2 western blot of proteins used in subsequent panels. **C**. The Tpm1 ΔBP DMS2 minigene was transfected alone (lane 1), or co-transfected with MS2 (lane 17), MS2 fused to full-length MBNL1 (lane 2), or the various deletion constructs indicated by the amino acid coordinates. Δ116-183 (lane 4) is a natural MBNL1 isoform resulting from exon skipping. Lanes 14–16 show the effects of the ZF1 and 2 mutations in the context of the 2-116-MS2 deletion mutant. In the experiment shown, the 2-253-MS2 was not expressed (lane 3); in other experiments (e.g. Figure 2E) its activity was similar to FL-MBNL1. The asterisked band is an artefactual band that does not appear in most experiments. Values significantly different from wild type MBNL1-FL-MS2 in panels C and D: **, P < 0.01; *** P < 0.001; ns, not significant. **D**. The Vldlr-MS2 minigene was co-transfected with the same effectors as panel C. **E**. Comparison of effects of mutations in panels C and D. The histogram indicates “% activity” for each of the MBNL1 constructs with the Tpm1 (green bars) and Vldlr (red bars) substrates. The values shown are relative to the activity of FL-MBNL1-MS2. 100% activity is defined by the difference in exon skipping/inclusion in the presence of FL-MBNL1-MS2 (upper horizontal line in histograms of panels C and D) and in the absence of co-transfection (lower horizontal lines). The comparison shows more rapid decline of activation than repression with C-terminal deletions into the ZF23 linker.

To analyse further the roles of the pairs of tandem zinc fingers we tested a series of deletion mutations based on MBNL-N-MS2. These included a C-terminal deletion series (previously tested on Tpm1 [22]), a natural deletion variant lacking the C-terminal half of the linker (Δ116-183), an N-terminal deletion series, and the linker alone. The linker sequence is predicted to be unstructured, but parts of it are highly conserved and have been shown previously to have a role in MBNL activities [17,18,22]. We expressed these proteins as MS2-fusions (Figure 6B) and analysed their activity when recruited to either the downstream Tpm1 (repressed, Figure 6C) or Vldlr (activated, Figure 6D) sites.

When recruited downstream of the MBNL-repressed Tpm1 exon 3 (the Tpm1-ΔbpDMS2 minigene) zinc fingers 3 and 4 and the C-terminal part of the linker could be removed individually or in combination with no effect (Figure 6C, lanes 4–6). C-terminal truncations beyond amino acid 116 led to diminished activity (lanes 6–9). Complete removal of the linker sequence and an alpha-helix of zinc finger 2 leaving only the first two zinc fingers results in an inactive protein (lane 9). Despite the importance of the linker region, when recruited alone it had no activity above MS2 alone (lane 10, 17). Zinc fingers 3 and 4 along with the complete preceding linker region, or with just the C-terminal part of the linker, were partially active (lanes 11,12). However, this activity required the linker sequence as ZF34 alone were inactive (lane 13). These data show that the N-terminal part of the protein comprising amino acids 2–102, encompassing the first two zinc fingers plus a third of the linker sequence, constitutes a minimal repressor domain. Introduction of RNA binding mutations into ZF1 or 2 individually drastically reduced activity of the 2–116 repressor domain, while combined mutation of ZF 1 and 2 abolished activity (compare lanes 14–16 with lane 6).

A similar, but not identical, response to the mutations was seen in the Vldlr context (Figure 6D). In this case, more of the linker sequence was required for full activity with progressively diminishing activity upon C-terminal deletion into the linker (Figure 6D, lanes 5–9, Figure 6E). Deletion of the second pair of zinc fingers (lane 5) or internal deletion of the C-terminal part of the linker (lane 4) had no effect on activity. As for repressor activity, the linker sequence alone was inactive (lane 10), but in combination with the second pair of zinc fingers the fusion protein retained substantial activity (lane 11), albeit less than the first pair (lane 5). This activity was reduced further by removal of the N-terminal part of the linker (lane 12) and abolished when only ZF34 remained (lane 13). Thus splicing activation is more dependent than repression upon the full linker sequence (Figure 6E). Although the activity of the 2-116 construct was already diminished, we tested the importance of RNA binding. Abrogating the RNA binding capacity of the 116 construct led to a severe, albeit not total reduction in activity (Figure 6D, lanes 14–16 compared to 6).

We analysed RNA binding of some of the C-terminal deletion fragments (Figure 5D-F). The miminal repressor domain MBNL1-2-116 bound to the Vldlr and Tpm1 RNAs with affinity reduced compared to the complete N-terminus but actually higher than the N-terminus with point mutations in ZF34 (Figure 5D compared to 5A,B). In addition, the 2–116 protein showed additional subsequent binding events on all three RNAs, consistent with the fact that it has only 2 ZFs that can contact the RNA. The 2–91 protein, which showed almost complete loss of tethered activation activity (Figure 5D lanes 6–8) bound to each RNA with affinity similar to 2–116 (Figure 5E), emphasizing that RNA binding is necessary but not sufficient for activity. Finally, the 2–72 protein, which lacks an experimentally observed C-terminal extension to the ZF2 domain [12,13] failed to bind RNA at any concentration, confirming the importance of the additional α-helix (Figure 5F).

### PTB associates with Vldlr RNA but does not regulate its splicing

**Figure 7:**
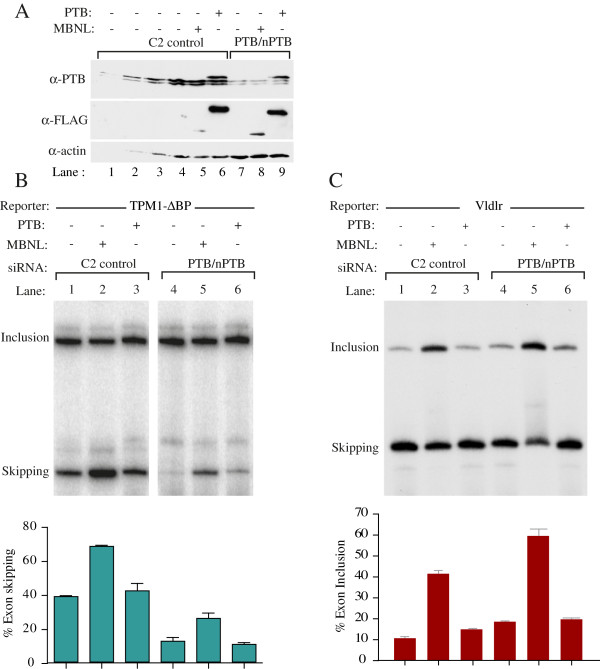
**PTB co-regulates Tpm1 but not Vldlr splicing. A**. Western blots with anti-PTB (upper), anti-FLAG (middle) and anti-actin (lower panel). Cells were treated with control C2 siRNA (lanes 1–6) or PTB/nPTB siRNAs (lanes 7–9). Lanes 4–1 show successive 2-fold dilutions of the C2 control to allow assessment of knockdown. In lanes 6 and 9, FLAG-PTB was overexpressed; in lanes 5 and 8, FLAG-MBNL1 was overexpressed. **B**. The Tpm1 ΔBP minigene was cotransfected with control C2 (lanes 1–3) or PTB/nPTB siRNAs (lanes 4–6). In addition, FLAG-PTB (lanes 3, 6) or FLAG-MBNL1 (lanes 2, 5) were also cotransfected. **C**. The Vldlr minigene was cotransfected with control C2 (lanes 1–3) or PTB/nPTB siRNAs (lanes 4–6). In addition, FLAG-PTB (lanes 3, 6) or FLAG-MBNL1 (lanes 2, 5) were also cotransfected.

MBNL and PTB act as co-repressors of Tpm1 splicing [22]. Pull-downs with biotinylated Vldlr RNA indicated that PTB was one of the major binding proteins in HeLa nuclear extract (data not shown). We therefore asked whether PTB acted synergistically or antagonistically with MBNL1 in the regulation of Vldlr splicing. As shown earlier, overexpression of MBNL1 promoted skipping of Tpm1 exon 3 but inclusion of the Vldlr exon (Figure 7B, C lanes 1,2). Overexpression of PTB had little effect on Tpm1 splicing (Figure 7C lane 3), as PTB is not limiting in HeLa cells [29]. However, PTB/nPTB knockdown led to decreased exon skipping (lanes 1 and 4). In contrast, Vldlr splicing was unresponsive to either overexpression or knockdown of PTB (Figure 7C lanes 1,3,4), suggesting that binding of PTB to Vldlr is non-functional. Furthermore, the activating effect of MBNL was not reduced upon PTB knockdown, and actually appeared to be slightly increased (Figure 7C lane 5). Therefore, while MBNL1 and PTB cooperate to repress Tpm1 splicing, MBNL1 acts independently to activate Vldlr splicing, and PTB binding to Vldlr appears to be non-functional.

## Discussion

The data presented here, drawing upon point mutations to impair RNA binding of ZF domains, deletion mutations, and artificial MS2 tethering, provide insights into the domains of MBNL1 that are involved in activation and repression of splicing. Our results are complementary to other published reports [16-18], and taken together the different studies converge upon some common themes. Among our key findings are the following. First, the N-terminal region of MBNL1 encompassing the four ZF domains is nearly fully active in most assays (Figure 2). Second the C-terminal region is inactive in conventional overexpression assays, but retains some activity in tethered assays where the MS2 domain recruits it to target RNAs (Figure 2). This residual activity might be associated with the ability of the C-terminal region to mediate dimerization [16,28], which might allow tethered C-terminal to interact with intact endogenous MBNL proteins, perhaps promoting their recruitment to the RNA. Third, MBNL1 is remarkably tolerant of RNA binding mutations to individual ZF domains (Figures 1, 3), consistent with previous results [18]. Fourth, pair-wise inactivation of ZF12 was in most cases more deleterious than inactivation of ZF34 (Figure 3). This is consistent with the effects of deletions that remove ZF12 or ZF34 (Figure 6, constructs 72–253 and 2–183 respectively), and with the effects of the didomain point mutations upon RNA binding (Figure 5A-C). Fifth, both repression of the Tpm1 exon and activation of the Vldlr exon required not just an intact ZF didomain, but also the linker sequence connecting ZF2 and 3 (Figure 6). Finally, there were some differences in the responses of the repressed Tpm1 and the activated Vldlr exons to different MBNL1 mutations. Activation of the Vldlr exon was more sensitive than repression of Tpm1 to deletions of the linker between ZF2 and 3 (Figure 6), consistent with previous reports comparing activated and repressed exons [16,17]. The minimal repressor domain for MBNL1 when tethered encompasses zinc fingers 1 and 2, with a region of further linker sequence up to amino acid 102. The minimal tethered activation domain comprises ZF1 and 2 and the full linker sequence to amino acid 183. In contrast to the effects of truncation mutations, effects of the ZF12 RNA binding mutations were more pronounced upon Tpm1 than Vldlr. The ZF12 mutation led to complete loss of activity upon the Tpm1 exon, whereas with the Vldlr minigene this mutation led to only a slight reduction in activity (Figure 3). Likewise, within the context of the effector fragment 2-116-MS2, mutation of ZF12 abolished repressor activity, while retaining ~30% of activator activity (Figure 6). The differential effects of MBNL1 mutations upon the Tpm1 and Vldlr exons are interesting. However, they cannot be generalized for all activated or repressed targets of MBNL proteins. Indeed, a systematic analysis of combined MBNL1 ZF mutants with a panel of 6 MBNL regulated events, showed that the relationship between ZF mutations and effects upon activity upon different splicing substrates is quite complex, with at least two classes of target, each encompassing repressed and activated targets [18]. In agreement with our conclusions the same study showed that MBNL activity upon Vldlr did not correlate well with its RNA binding ability [18].

A surprising feature of our results is that the ZF RNA binding mutations had a greater effect in the MS2 tethering assays (Figure 3) than in the untethered assays (Figure 1). One would anticipate that the tethered proteins would be less sensitive to mutations in their RNA binding domains, since the MS2 domain should bypass at least some of the RNA binding functions of the intact protein. However, the C-terminal domain, which mediates dimerization [16,28] and on its own has some activity in the tethering assays (Figure 2), was missing in most of the subsequent MS2-tethering assays (Figures 3, 6), which might account for the greater sensitivity to the ZF mutations. In addition to the RNA binding mutations of the ZF domains, we also tested a set of mutations in ZF4 designed to impair intra-molecular contacts formed between MBNL molecules in crystal structures. Although previous studies have implicated the variably spliced C-terminal region of MBNL in dimerization [16,28], it was possible that the ZF4 mediated contacts might also be functional. However, these mutations had no functional effect in a number of assays of MBNL1 activities (Figure 4). Although we did not test the mutations in direct assays for dimerization, the lack of effect in functional assays suggests that these contacts are not physiologically relevant.

The deleterious effects of RNA binding mutations in a tethered function assay is initially surprising, given that the starting point of the assay is to bypass the normal mode of RNA binding at a particular location. However, this has been observed with similar studies of RbFOX [23] and PTB [30], and provides insights into the possible mechanisms of splicing regulation. Given that many RNA binding proteins, including MBNL proteins, have multiple RNA binding domains, it could be that the MS2 tether replaces the role of a subset of the RNA binding domains, and that functional effects require the remaining domains to interact with RNA for one of a number of reasons. The protein might need to bind to more than one site in the target pre-mRNA to be functional, perhaps forming an RNA architecture conducive to regulated exon skipping or inclusion; the protein might have to interact with another RNA; or the protein might have to interact with RNA in order to interact with important partner proteins. Taken in order, we already know that there are at least two major MBNL1 binding sites flanking Tpm1 exon 3 [22,31]. We also identified additional YGCY motifs downstream of the Vldlr exon that mediate MBNL1 activity (data not shown). Alternatively, the additional RNA interactions could be with a distinct RNA species such as U1 snRNA; inhibition of the N1 exon of *CSRC* involves a PTB-U1 snRNA interaction [32]. Finally, RNA binding might be important to induce a conformation that facilitates interaction with co-regulatory proteins [22]. These different explanations are not all mutually exclusive; for example, a protein-RNA interaction might promote a necessary RNP architecture as well as inducing a conformation change promoting necessary protein-protein interactions.

Analysis of the relationship between position of binding on RNA substrates and the mode of action as a repressor or activator indicates that in general MBNL binding upstream on an exon is associated with repression while downstream binding leads to activation [10,33,34], similar to a number of other regulatory proteins [4]. However, in the case of repression there are also binding peaks downstream of the exon as well, suggesting a sub-set of events which are regulated negatively by MBNL sites flanking the exon. This suggests the possibility of two discrete types of MBNL-repressed event, which might operate in a mechanistically distinct manner or might share some common mechanistic elements. In the first case, MBNL binding sites immediately upstream of the regulated exon might be sufficient to interfere with binding or activity of constitutive splicing factors such as U2AF, as has been suggested in the cTNT transcript [35,36]. In the case of exons such as Tpm1 exon 3 the flanking sites might be necessary in order for the upstream sites to effectively interfere with 3′ splice site recognition factors, perhaps by cooperative binding of oligomers and consequent looping of intervening RNA [37,38]. Alternatively the flanking regulatory sites might need to act in a concerted way on the 3′ and 5′ splice sites. The two tandem zinc finger arrays of MBNL are arranged with each zinc finger ‘back-to-back’, which would cause an anti-parallel alignment of a bound RNA. It appears unlikely that a single MBNL1 protein could bind to both the elements flanking Tpm1 exon 3. Indeed, mutations in ZF12 and ZF34 have similar effects upon binding to either the upstream or the downstream Tpm1 element (Figure 5A-C). However, we have recently shown that the minimal repressor region of MBNL interacts with PTB protein, in an RNA-dependent manner [22], and there are PTB sites flanking exon 3, with two to three molecules binding either side [39]. Moreover MBNL has been shown to interact with itself [16,28]. Taken together this suggests a complex forming across the exon, with homotypic and heterotypic interactions between MBNL and PTB molecules acting to stabilise a looped structure which promotes exon skipping. Analysis of proteins binding to the Vldlr substrate indicated strong interaction of PTB, suggesting that it might act as a coactivator. However, overexpression and knockdown experiments clearly showed that PTB played no role in regulating Vldlr splicing (Figure 7). Indeed, we also found that PTB-MS2 had no activity when tethered downstream of the Vldlr exon, despite the fact that it can activate its own target exons from this location [40]. This clearly indicates that different activators have distinct molecular targets, even when binding at similar locations. An important future line of work will be to identify the molecular targets of the minimal MBNL1 activator domain.

## Methods

### Constructs

The *Tpm1* minigene reporters and Δbp mutation have been described previously [22,27,31]. The Vldlr wildtype minigene was a kind gift from Prof. Manny Ares (University of California Santa Cruz) [10]. This minigene was mutagenized to introduce a Pst1 site then an MS2 hairpin was inserted using the following oligo: 5’-gAGGATCACCctgca-3’. Expression plasmids for MBNL1 N and C terminal truncations have been described previously [22]. Mutagenesis was performed using standard protocols and the following primers: ZnF1: 5-CACGGAATGTAAAgcTGCACATCCTTCG-3, ZnF2: 5-GGAGAACTGCAAAgcTCTTCATCCACC-3, ZnF3: 5-GAAAATGATTGTCGGgcTGCTCATCCTGC-3, ZnF4: 5-GGAAAAGTGCAAAgcCTTTCATCCCCC-3. MBNL1 truncations were cloned by inserting the coding sequence for the relevant portion of MBNL1 into the AvrII and MluI sites in the pCIMS2-NLS-FLAG vector [20,41]. Plasmids for bacterial expression were cloned by insertion of appropriate coding sequence into the pGEX-4-T3 vector. Sequences for expression of Tpm1 or Vldlr RNA were cloned into pGEM-4Z plasmids (Promega), and transcribed usingT7 polymerase. Sequences used were, Vldlr: GGGAGACAAGCTTTGCAAACTGTTAATCTCAACTAACTGCCGCTTAAATAATTAGTGCAGCTTTTAACTACTGGTTCTGTCCCAACTGGCTACTTGTGCCTAAAGCCCAAAGAATT, Dugc: GGGAGACAAGCTTGAGCTGGATGCCGCCTCTGCTGCTGC, URE: gggagacaagcttaaGTCTACGCACCCTCAAccCGCACCTTGCGGGATCACGCTGCCTGCTGCACCCCACCCCCTTCCCCCTTCCTTCCCCCCACCCCCGTACTCCACTGCCAACTCCCAG.

### Cell culture

Cells were transfected a day after splitting to 10^5^ – 2 × 10^5^ cells per well in a 6 well plate. Transfections were performed using 400 ng effector construct unless otherwise stated, with 200 ng reporter, made up to 1 μg with empty pGEM4Z vector as necessary. Per well 1μg of DNA, 100 μl Optimem and 2 μl Lipofectamine (Invitrogen 18324–012) was used. Lipofectamine-DNA mix was incubated for 30min at room temperature, then diluted to 1ml in Opti-MEM-1 and applied to cell monolayer previously washed with PBS. Treated HeLa cells were incubated for 5 hr at 37°C, and then the transfection mix was replaced with 2 ml Dulbecco’s Modified Eagles Medium (DMEM) supplemented with Glutamax & 10% fetal bovine serum (FBS). Cells were then incubated for a further 48 hours, then RNA and protein was harvested from the cultures using Trizol reagent (invitrogen) or boiled SDS loading buffer respectively.

For MBNL1 knockdown, the following target sequence was used: 5’-AACACGGAAUGUAAAUUUGCA-3’ [42]. HeLa cells were split to a density of 2 × 10^5^ in 1.7 ml DMEM +10% FBS medium in 6 well plates, and incubated at 37°C for 24 hours. Each well was treated with 10 nM siRNA (THH2 siRNA for MBNL1 knockdown or control C2 siRNA) and 15 μl Oligofectamine (Invitrogen 12252–011), diluted in 500 μl Optimem and incubated prior at room temperature for 20 minutes. Cells were then incubated for 24 hours at 37°C. After 24 hour incubation DNA transfections were performed as above using lipofectamine or lipofectamine 2000 reagent. Cells were incubated for 5 hours at 37°C, then the medium on them replaced with 1.5ml DMEM + 10% FBS. To each well 10 nM siRNA and 3 μl Lipofectamine 2000 reagent in 500 μl Optimem was added, which had been pre-incubated for 20 minutes at room temperature. Cells were incubated for a further 48 hours, then harvested.

### RNA and protein analysis of transfections

For RNA analysis, cells in 6 well plates were washed using twice with 2 ml PBS, then to each well 1 ml tri-reagent (Sigma-Aldrich) was added, and purified according to the manufacturers’ protocols. Samples were DNAse I treated using 2 units of rDNAse (Ambion) in 10 mM Tris pH 7.5, 2.5 mM MgCl_2_, 0.5 mM CaCl_2_ for 30 minutes – 1 hour at 37°C, phenol extracted and ethanol precipitated. PCR analysis used the following primers for Vldlr minigenes:

V4rt - 5’-GTGGCAAAGGTGCCCTTGAG-3’ - (rt primer)

V1 - 5’-ACGTGGATGAAGTTGGTGGT-3’ - (5’ primer)

V3 - 5’-GGCACCGAGCACTTTCTTGC-3’ - (3’ primer)

and the following primers for Tpm1 minigenes:

SV3’RT: 5’-GCAAACTCAGCCACAGGT-3’ - (rt primer)

SV5’2: 5’-GGAGGCCTAGGCTTTTGCAAAAAG-3’ - (5’ primer)

SV3’1: 5’-ACTCACTGCGTTCCAGGCAATGCT-3’ - (3’ primer)

Reverse transcriptions were performed using 2–3 μg of total RNA, and 100 ng of RT primer, in 50 mM Tris pH 6.3, 40 mM KCl, 8 mM MgCl2, 2 mM DTT. Samples were heated for 15 minutes at 55°C, then cooled to 42°C, and 2 μl 10 mM dNTP and 1 μl AMV-RT (Promega) enzyme added, and incubated at 42°C for 60 minutes. For the PCR reaction, the 3’ primer was 5’ end labeled with [^32^P]-ATP,. Oligo primer (4 pmoles per PCR reaction) was incubated at 37°C for 60 minutes in 50 mM Tris 10 mM MgCl_2_ T4 polynucleotide kinase enzyme (NEB) and 0.1 μl [α-^32^P]-UTP per PCR reaction. After incubation the solution was phenol extracted, and purified on a G-50 spin column (GE Healthcare). The labelled oligo was made up to concentration of 1 pmole/μl. 2 μl of RT reaction was taken into fresh eppendorf in buffer (50 μM KCl, 10 μM Tris pH 8.3, 1.5 mM MgCl2, 0.001% w/v gelatin) and 25 pmole of the reverse primer. Samples were heated to 92°C for 3 minutes, then cooled to 80°C, and 0.25 μl Taq polymerase (Roche) and 10 pmol ^32^P-labeled probe added. The samples were then cycled for 30 cycles of 94°C for 30 seconds, 62°C for 30 seconds and 72°C for 60 seconds. RT-PCR products were analysed on denaturing 4% PAGE gels, using Sequagel (National Diagnostics EC-833) system. Samples were diluted in formamide loading buffer, heated to 90°C for 5 minutes, then loaded. Gels were run for 100 minutes at constant 38 W, the gel was dried and exposed on phosphorimager casette (Molecular Dynamics). The results were quantified using ImageQuant Software (GE Healthcare) and analysed using Excel (Microsoft) and Graphpad 5 (Prism Software). Statistical judgements were made using either students t-tests or, where multiple combinations tested, a post-ANOVA Tukey test, which is a variation of the students t-test which aims to eliminate type 2 errors stemming from multiple comparisons without Bonferroni corrections. Statistical significance is indicated by: ns, not significant; * P < 0.05; ** P < 0.01; ***, P < 0.001.

For protein analysis, the cell monolayer in 6 well plates was washed twice with 2 ml PBS, then directly to each well 150 μl of hot SDS buffer (pre-heated to 100°C for 5 minutes) was added. The cells were scrapped using upturned P-1000 tips, extracted into eppendorf tubes, and frozen on dry ice. Samples were heated again to 100°C for 5 minutes, separated using SDS-PAGE, analysed using standard western blotting techniques, and imaged using standard ECL techniques. For western blot analysis primary antibodies were in house anti-rabbit MS2 or anti-FLAG from Sigma (F1804). Protein loading was checked by Ponceau staining and, in some cases, by re-probing with anti-actin antibodies.

### Recombinant protein expression

Recombinant MBNL1 protein was expressed and purified from *E.coli* BL21 cells. 400 ml cultures were induced at OD_600_ = 0.5 by the addition of 1 mM IPTG, and grown for 3 hours shaking at 225 rpm. The cultures were then pelleted, washed in MTPBS (150 mM NaCl, 16 mM Na_2_HPO_4_, 4 mM NaH_2_PO_4_, pH 7.3) and lysed using a French Press (Stansted Fluid Power) according to the manufacturer’s instructions. The homogenised samples were centrifuged at 7741 rcf, 4°C, for 10 minutes. Samples were then purified using GST Sepharose 4B beads (GE Healthcare) according to manufacturers protocols. Briefly - the beads were pre-washed with 5–10 volumes of water, MTPBS and MTPBS + 1% Triton-X100. To the homogenised sample Triton-X100 added to concentration of 1%. This bacterial homogenate was then incubated with the GST beads at 4°C, for 1 hour. The beads were washed 4 times with 2.5 volumes of MPTBS + 1% Triton-X100, then loaded into a disposable biorad column at 4°C. The recombinant proteins were eluted from the column using 3×800 μl of 25 mM reduced glutathione in 100 mM HEPES (pH 8.9), followed by 3×800 μl of 50 mM reduced glutathione in 100 mM HEPES. All fractions of interest were pooled, and dialysed in 1.8 litres of Dignam Buffer E overnight at 4°C using Slide-A-Lyzer Dialysis Cassettes (Thermo Scientific), according to manufacturer’s protocols. The concentration of the recombinant proteins was estimated with reference to BSA standards.

### Electrophoretic-mobility shift and UV crosslinking assay

High specific activity [α-P^32^] UTP labelled RNAs were made using standard protocols with either SP6 or T7 polymerase. Binding reactions were set up in microtitre plates (Corning) pre-lined with BSA. Mobility shift assays had a total 5 μl reaction volume, with 10 fmol RNA, 20 μg/ml rRNA, 10 mM HEPES pH 7.9, 10 μM ZnCl2, 3 mM MgCl2, 5% Glycerol, 1 mM DTT, and proteins at appropriate concentration. The reaction was incubated for 15 minutes at 30°C, then 0.5 μl of a 55 mg/ml Heparin (Sigma) added, and the samples incubated for a further 5 minutes. Before loading onto the gel 1 μl of 50% glycerol was added. 5% poly-acrylamide gels were used, with 30:1 bis:acrylamide ratio. Gels were run at 200 volts for ~ 2hr, 4°C after pre-running for 1–2 hours, at 200 volts. Dried gels were analysed by phosphor-imager (Molecular Dynamics). After scanning on Typhoon scanner results were analysed using ImageQuant (GE Healthcare) and Photoshop (Adobe). Dissociation constants were estimated from the total protein concentration that produced 50% binding. For UV crosslinking, the total reaction volume was 10 μl. After addition of heparin, samples were subjected to 19200 J.cm^-2^ UV light, followed by digestion with 50 μg RNase A and 140 U RNase T1. Samples were then separated by SDS gel electrophoresis and dried gels analyzed by phosphorimager.

## Conclusions

Our results highlight the common and distinct domain requirements for activation or repression of splicing by MBNL1. Full length MBNL1 is relatively insensitive to inactivating mutations of individual ZF domains. However, when the protein is recruited to RNA by tethering with a heterologous RNA binding domain and deletion mutations are introduced, the dependency on functional ZF domains becomes more acute. Full tethered repressor and activator functions require ZF domains 1 and 2 that are able to bind RNA, suggesting that both types of activity require multivalent interactions with RNA. However, the ZF domains alone are insufficient for activity. Additional regions of the linker separating ZF domains 2 and 3 are required for splicing activity but not RNA binding. The additional regions differ for repression or activation, with more extensive regions of the linker required for full activation. This suggests the involvement of different sets of interacting cofactors for activation or repression of splicing by MBNL1.

## Competing interest

The authors declare that they have no competing interests.

## Authors’ contributions

CWJS and CG conceived of the study and participated in its design and coordination. CE and CG carried out the experimental work and analyzed the data. All authors helped to draft the manuscript, and approved the final manuscript.

## References

[B1] Nilsen TW Graveley BR Expansion of the eukaryotic proteome by alternative splicing Nature 2010 14 7280 457 463 10.1038/nature08909 20110989 PMC3443858

[B2] Barbosa-Morais NL Irimia M Pan Q Xiong HY Gueroussov S Lee LJ Slobodeniuc V Kutter C Watt S Colak R The evolutionary landscape of alternative splicing in vertebrate species Science 2012 14 6114 1587 1593 10.1126/science.1230612 23258890

[B3] Merkin J Russell C Chen P Burge CB Evolutionary dynamics of gene and isoform regulation in Mammalian tissues Science 2012 14 6114 1593 1599 10.1126/science.1228186 23258891 PMC3568499

[B4] Witten JT Ule J Understanding splicing regulation through RNA splicing maps Trends Genet 2011 14 3 89 97 10.1016/j.tig.2010.12.001 21232811 PMC3165201

[B5] Miller JW Urbinati CR Teng-Umnuay P Stenberg MG Byrne BJ Thornton CA Swanson MS Recruitment of human muscleblind proteins to (CUG) (n) expansions associated with myotonic dystrophy EMBO J 2000 14 17 4439 4448 10.1093/emboj/19.17.4439 10970838 PMC302046

[B6] Kalsotra A Xiao XS Ward AJ Castle JC Johnson JM Burge CB Cooper TA A postnatal switch of CELF and MBNL proteins reprograms alternative splicing in the developing heart Proceedings of the National Academy of Sciences of the United States of America 2008 14 51 20333 20338 10.1073/pnas.0809045105 19075228 PMC2629332

[B7] Terenzi F Ladd AN Conserved developmental alternative splicing of muscleblind-like (MBNL) transcripts regulates MBNL localization and activity RNA Biol 2009 14 1 43 55 10.4161/rna.7.1.10401 20009516

[B8] Botta A Caldarola S Vallo L Bonifazi E Fruci D Gullotta F Massa R Novelli G Loreni F Effect of the [CCTG]n repeat expansion on ZNF9 expression in myotonic dystrophy type II (DM2) Biochim Biophys Acta 2006 14 3 329 334 10.1016/j.bbadis.2005.11.004 16376058

[B9] Osborne RJ Lin X Welle S Sobczak K O’Rourke JR Swanson MS Thornton CA Transcriptional and post-transcriptional impact of toxic RNA in myotonic dystrophy Human Molecular Genetics 2009 14 8 1471 1481 10.1093/hmg/ddp058 19223393 PMC2664149

[B10] Du H Cline MS Osborne RJ Tuttle DL Clark TA Donohue JP Hall MP Shiue L Swanson MS Thornton CA Aberrant alternative splicing and extracellular matrix gene expression in mouse models of myotonic dystrophy Nat Struct Mol Biol 2010 14 2 187 193 10.1038/nsmb.1720 20098426 PMC2852634

[B11] Kino Y Washizu C Oma Y Onishi H Nezu Y Sasagawa N Nukina N Ishiura S MBNL and CELF proteins regulate alternative splicing of the skeletal muscle chloride channel CLCN1 Nucleic Acids Res 2009 14 19 6477 6490 10.1093/nar/gkp681 19720736 PMC2770659

[B12] He F Dang W Abe C Tsuda K Inoue M Watanabe S Kobayashi N Kigawa T Matsuda T Yabuki T Solution structure of the RNA binding domain in the human muscleblind-like protein 2 Protein Sci 2009 14 1 80 91 19177353 10.1002/pro.17 PMC2708044

[B13] Teplova M Patel DJ Structural insights into RNA recognition by the alternative-splicing regulator muscleblind-like MBNL1 Nat Struct Mol Biol 2008 14 12 1343 1351 10.1038/nsmb.1519 19043415 PMC4689322

[B14] Goers ES Purcell J Voelker RB Gates DP Berglund JA MBNL1 binds GC motifs embedded in pyrimidines to regulate alternative splicing Nucleic Acids Res 2010 14 7 2467 2484 10.1093/nar/gkp1209 20071745 PMC2853123

[B15] Cass D Hotchko R Barber P Jones K Gates DP Berglund JA The four Zn fingers of MBNL1 provide a flexible platform for recognition of its RNA binding elements BMC Mol Biol 2011 14 20 10.1186/1471-2199-12-20 21548961 PMC3103431

[B16] Tran H Gourrier N Lemercier-Neuillet C Dhaenens CM Vautrin A Fernandez-Gomez FJ Arandel L Carpentier C Obriot H Eddarkaoui S Analysis of exonic regions involved in nuclear localization, splicing activity, and dimerization of Muscleblind-like-1 Isoforms Journal of Biological Chemistry 2011 14 18 16435 16446 10.1074/jbc.M110.194928 21454535 PMC3091249

[B17] Grammatikakis I Goo YH Echeverria GV Cooper TA Identification of MBNL1 and MBNL3 domains required for splicing activation and repression Nucleic Acids Res 2011 14 7 2769 2780 10.1093/nar/gkq1155 21109529 PMC3074124

[B18] Purcell J Oddo JC Wang ET Berglund JA Combinatorial Mutagenesis of MBNL1 Zinc Fingers Elucidates Distinct Classes of Splicing Regulatory Events Mol Cell Biol 2012 14 20 4155 4167 10.1128/MCB.00274-12 22890842 PMC3457334

[B19] Graveley BR Maniatis T Arginine/serine-rich domains of SR proteins can function as activators of pre-mRNA splicing Mol Cell 1998 14 5 765 771 10.1016/S1097-2765(00)80076-3 9660960

[B20] Del Gatto-Konczak F Olive M Gesnel MC Breathnach R hnRNP A1 recruited to an exon in vivo can function as an exon splicing silencer Mol Cell Biol 1999 14 1 251 260 9858549 10.1128/mcb.19.1.251 PMC83883

[B21] Robinson F Smith CW A splicing repressor domain in polypyrimidine tract-binding protein J Biol Chem 2006 14 2 800 806 16282332 10.1074/jbc.M510578200

[B22] Gooding C Edge C Lorenz M Coelho MB Winters M Kaminski CF Cherny D Eperon IC Smith CW MBNL1 and PTB cooperate to repress splicing of Tpm1 exon 3 Nucleic Acids Res 2013 14 9 4765 4782 10.1093/nar/gkt168 23511971 PMC3643581

[B23] Sun S Zhang Z Fregoso O Krainer AR Mechanisms of activation and repression by the alternative splicing factors RBFOX1/2 Rna 2011 14 2 274 283 22184459 10.1261/rna.030486.111 PMC3264914

[B24] Shankarling G Lynch KW Minimal functional domains of paralogues hnRNP L and hnRNP LL exhibit mechanistic differences in exonic splicing repression Biochem J 2013 14 2 271 279 10.1042/BJ20130432 23646903 PMC4069513

[B25] Hudson BP Martinez-Yamout MA Dyson HJ Wright PE Recognition of the mRNA AU-rich element by the zinc finger domain of TIS11d Nat Struct Mol Biol 2004 14 3 257 264 10.1038/nsmb738 14981510

[B26] Fu Y Ramisetty SR Hussain N Baranger AM MBNL1-RNA Recognition: Contributions of MBNL1 Sequence and RNA Conformation Chembiochem 2012 14 1 112 119 10.1002/cbic.201100487 22106026 PMC3890438

[B27] Gooding C Clark F Wollerton MC Grellscheid SN Groom H Smith CW A class of human exons with predicted distant branch points revealed by analysis of AG dinucleotide exclusion zones Genome Biol 2006 14 1 R1 10.1186/gb-2006-7-1-r1 16507133 PMC1431707

[B28] Yuan Y Compton SA Sobczak K Stenberg MG Thornton CA Griffith JD Swanson MS Muscleblind-like 1 interacts with RNA hairpins in splicing target and pathogenic RNAs Nucleic Acids Res 2007 14 16 5474 5486 10.1093/nar/gkm601 17702765 PMC2018611

[B29] Wollerton MC Gooding C Robinson F Brown EC Jackson RJ Smith CW Differential alternative splicing activity of isoforms of polypyrimidine tract binding protein (PTB) Rna 2001 14 6 819 832 10.1017/S1355838201010214 11421360 PMC1370133

[B30] Joshi A Coelho MB Kotik-Kogan O Simpson PJ Matthews SJ Smith CW Curry S Crystallographic analysis of polypyrimidine tract-binding protein-Raver1 interactions involved in regulation of alternative splicing Structure 2011 14 12 1816 1825 10.1016/j.str.2011.09.020 22153504 PMC3420021

[B31] Gromak N Smith CW A splicing silencer that regulates smooth muscle specific alternative splicing is active in multiple cell types Nucleic Acids Res 2002 14 16 3548 3557 10.1093/nar/gkf480 12177296 PMC134246

[B32] Sharma S Maris C Allain FH Black DL U1 snRNA directly interacts with polypyrimidine tract-binding protein during splicing repression Mol Cell 2011 14 5 579 588 10.1016/j.molcel.2011.02.012 21362553 PMC3931528

[B33] Wang ET Cody NA Jog S Biancolella M Wang TT Treacy DJ Luo S Schroth GP Housman DE Reddy S Transcriptome-wide regulation of pre-mRNA splicing and mRNA localization by muscleblind proteins Cell 2012 14 4 710 724 10.1016/j.cell.2012.06.041 22901804 PMC3428802

[B34] Charizanis K Lee KY Batra R Goodwin M Zhang C Yuan Y Shiue L Cline M Scotti MM Xia G Muscleblind-like 2-mediated alternative splicing in the developing brain and dysregulation in myotonic dystrophy Neuron 2012 14 3 437 450 10.1016/j.neuron.2012.05.029 22884328 PMC3418517

[B35] Warf MB Berglund JA MBNL binds similar RNA structures in the CUG repeats of myotonic dystrophy and its pre-mRNA substrate cardiac troponin T Rna 2007 14 12 2238 2251 10.1261/rna.610607 17942744 PMC2080590

[B36] Warf MB Diegel JV von Hippel PH Berglund JA The protein factors MBNL1 and U2AF65 bind alternative RNA structures to regulate splicing Proc Natl Acad Sci U S A 2009 14 23 9203 9208 10.1073/pnas.0900342106 19470458 PMC2695092

[B37] Nasim FU Hutchison S Cordeau M Chabot B High-affinity hnRNP A1 binding sites and duplex-forming inverted repeats have similar effects on 5’ splice site selection in support of a common looping out and repression mechanism Rna 2002 14 8 1078 1089 10.1017/S1355838202024056 12212851 PMC1370318

[B38] Fisette JF Toutant J Dugre-Brisson S Desgroseillers L Chabot B hnRNP A1 and hnRNP H can collaborate to modulate 5’ splice site selection Rna 2010 14 1 228 238 10.1261/rna.1890310 19926721 PMC2802032

[B39] Cherny D Gooding C Eperon GE Coelho MB Bagshaw CR Smith CW Eperon IC Stoichiometry of a regulatory splicing complex revealed by single-molecule analyses EMBO J 2010 14 13 2161 2172 10.1038/emboj.2010.103 20502437 PMC2905242

[B40] Llorian M Schwartz S Clark TA Hollander D Tan LY Spellman R Gordon A Schweitzer AC de la Grange P Ast G Position-dependent alternative splicing activity revealed by global profiling of alternative splicing events regulated by PTB Nat Struct Mol Biol 2010 14 9 1114 1123 20711188 10.1038/nsmb.1881 PMC2933513

[B41] Gromak N Rideau A Southby J Scadden AD Gooding C Huttelmaier S Singer RH Smith CW The PTB interacting protein raver1 regulates alpha-tropomyosin alternative splicing EMBO J 2003 14 23 6356 6364 10.1093/emboj/cdg609 14633994 PMC291850

[B42] Ho TH Charlet BN Poulos MG Singh G Swanson MS Cooper TA Muscleblind proteins regulate alternative splicing EMBO J 2004 14 15 3103 3112 10.1038/sj.emboj.7600300 15257297 PMC514918

